# The Role of Fecal Microbiota Transplantation in the Treatment of Neurodegenerative Diseases: A Review

**DOI:** 10.3390/ijms24021001

**Published:** 2023-01-05

**Authors:** Julie-Anne T. Matheson, R. M. Damian Holsinger

**Affiliations:** 1 Laboratory of Molecular Neuroscience and Dementia, School of Medical Sciences, Faculty of Medicine and Health, The University of Sydney, Camperdown, NSW 2050, Australia; 2 Neuroscience, School of Medical Sciences, Faculty of Medicine and Health, The University of Sydney, Camperdown, NSW 2006, Australia

**Keywords:** fecal microbiota transplant, neurodegenerative disease, Alzheimer’s disease, Parkinson’s disease, multiple sclerosis, amyotrophic lateral sclerosis, gut microbiome

## Abstract

Neurodegenerative diseases are highly prevalent but poorly understood, and with few treatment options despite decades of intense research, attention has recently shifted toward other mediators of neurological disease that may present future targets for therapeutic research. One such mediator is the gut microbiome, which communicates with the brain through the gut–brain axis and has been implicated in various neurological disorders. Alterations in the gut microbiome have been associated with numerous neurological and other diseases, and restoration of the dysbiotic gut has been shown to improve disease conditions. One method of restoring a dysbiotic gut is via fecal microbiota transplantation (FMT), recolonizing the “diseased” gut with normal microbiome. Fecal microbiota transplantation is a treatment method traditionally used for *Clostridium difficile* infections, but it has recently been used in neurodegenerative disease research as a potential treatment method. This review aims to present a summary of neurodegenerative research that has used FMT, whether as a treatment or to investigate how the microbiome influences pathogenesis.

## 1. Introduction

Neurodegenerative diseases present a unique challenge in the modern research landscape. Despite decades of research, our understanding of the causes and mechanisms underlying them is still limited, and very few effective treatments exist. Given the lack of progress, the focus of the research community is increasingly shifting to exploring new or alternative factors that could influence either the pathogenesis or treatment of these diseases. One such factor is the gut–brain axis, which encompasses the bidirectional interaction between the gut and brain through neural, immune, endocrine and metabolic channels [1,2,3,4]. Neurodegenerative diseases are often associated with abnormal gut microbiome compositions [5,6,7,8,9,10,11,12,13], and as such, it is not surprising that modifying the gut microbiome is quickly becoming an area of interest for future research.

An increasingly popular method of microbiome modification is fecal microbiota transplantation (FMT). This treatment involves the transfer of gut microbiota from a ‘healthy’ individual to one who has a “diseased” gut microbiome, typically with the goal of correcting dysbiosis in the recipient [14]. It is usually used to treat *Clostridium difficile* infection (CDI), for which it is remarkably effective [15]; however, it is increasingly being used to treat other gastrointestinal diseases [16]. In the past few years, it has emerged as an intriguing option for treating neurological disease as well, resulting in a rapidly growing pool of literature [17]. Therefore, this review aims to summarize the current research into neurodegenerative disease that involves FMT for study or treatment of four neurodegenerative diseases.

## 2. Alzheimer’s Disease

Alzheimer’s disease (AD) is the single most common form of dementia, affecting approximately 40 million individuals globally [18]. It is a progressive neurodegenerative disease known for gradual deterioration in cognitive ability, especially memory [19], and distinctive pathological characteristics such as amyloid-beta (Aβ) plaques [20,21,22], neurofibrillary tangles (NFTs) [20,21], and heightened neuroinflammation [23,24,25,26]. Alterations in the AD gut microbiome have been noted both in humans [27,28] and in animal models [29,30]; in fact, gut microbiome changes appear to *precede* neurological pathology in APP/PS1 [31] and ADLP^APT^ mice [29]. Probiotics [32] and antibiotics [33] have been associated with improvements in AD outcomes, suggesting that correcting gut microbiome abnormalities may be a viable target for AD treatment; since FMT involves more expansive modifications to the microbiome than either probiotics or antibiotics can achieve [16], FMT may be even more effective. Numerous lines of research investigating the efficacy of FMT in AD have been recently published and are summarized in Table 1.

### 2.1. Animal Research

The study of FMT in AD has revealed numerous intriguing findings. Sun and colleagues [34] investigated the effects of FMT in APP/PS1 mice, a widely characterized and extensively studied mouse model of the disease. Mice that were administered FMT from wild-type (WT) donors showed significant improvements in cognitive function, decreased Aβ plaque burden, and decreased levels of soluble Aβ_40_ and Aβ_42_ when compared to untreated littermates. Additionally, proteins associated with synaptic plasticity were upregulated, while within the gut there was a significant increase in the beneficial short-chain fatty acid (SCFA), butyrate [34].

In a 2019 pre-print published by Elangovan and colleagues [35], old 5xFAD mice, a well-characterized and aggressive mouse model of AD, were treated with FMT from either young (8–10-week-old) or age-matched (30–32-week-old) WT donors. All 5xFAD mice showed improvements in spatial memory and learning, alongside an overall reduction in amyloid plaque pathology. Interestingly, old transgenic mice that received FMT from young donors showed much stronger and significant improvements compared to those that received FMT from age-matched WT donors. This finding is supported by other research demonstrating that the transfer of FMT from old WT donors impairs spatial learning and memory in young WT recipients [36], while ageing-related symptoms are improved for old WT recipients of FMT from young WT donors [37].

FMT has also been used to demonstrate that the gut microbiome is intimately linked to AD pathogenesis, progression and severity. For example, Shen et al. [38] transplanted microbiota from AD patients into APP/PS1 mice to evaluate the influence of the gut microbiome on AD pathogenesis. They found that recipient mice performed significantly worse in behavioral measures and had increased neuroinflammation compared to transgenic littermates who did not receive FMT. Following this initial experiment, Shen and colleagues then treated a second group of APP/PS1 mice who had received FMT from AD patients with FMT from healthy human donors instead. Intriguingly, these mice showed improvements in memory and a reduction in inflammation across the gut, periphery and brain [38].

Further studies to demonstrate the involvement of the gut microbiome in AD pathogenesis, progression and severity examined the effects of transplanting microbial material from 5xFAD mice into WT recipients [39]. Behavioral tests and immunohistochemical analysis revealed significantly impaired memory function, reduced neurogenesis and increased inflammation in the brain and colon of WT recipients [39]. Fujii and colleagues reported that WT mice receiving FMT from a human AD patient developed cognitive deficits at an earlier age compared to littermates who were administered FMT from healthy human donors [40]. Kim et al. [29] also showed that long-term administration of FMT from WT donors into ADLP^APT^ mice resulted in decreased Aβ deposition, less soluble Aβ_40_ and reduced tau pathology in their brains [29]. 

Dodiya and coworkers have demonstrated that post-natal, long-term treatment of APPPS1-21 Tg mice with an antibiotic cocktail resulted in significantly decreased Aβ deposition [41]. Interestingly, these results were only observed in the male mice who also showed distinct alterations in peripheral cytokines/chemokines and changes in the composition of the fecal microbiome compared to conventionally raised littermates [41]. Contrastingly, antibiotic-treated female mice had a greater abundance of bacteria and metabolic pathways associated with pro-inflammatory phenotypes with increased levels of pro-inflammatory cytokines/chemokines in plasma, and no changes in Aβ load compared with non-treated controls [41]. Gut recolonization of the antibiotic-treated male mice with microbiota from APPPS1-21 Tg mice resulted in the reestablishment of an APPPS1-21 gut phenotype that was accompanied by Aβ amyloidosis and microglia changes similar to the donor phenotype [41]. Donor age is also an important factor that must be considered in fecal transplants, as shown by Elangovan and colleagues [35]. Similar results were reported by Wang and colleagues, who demonstrated that the colonization of three-month-old APP/PS1 mice, who had their innate microbiome depleted via antibiotics, with transplant material from 16-month-old APP/PS1 donors showed a significant increase in Aβ plaque formation when compared to age-matched littermates [42].

In a novel example of environmental influence on AD pathogenesis, Cui et al. [43] used senescence-accelerated mouse prone 8 (SAMP8) mice to explore the influence of sound (high- or low-intensity) on the gut microbiome. Mice exposed to high-intensity sound showed deterioration of cognitive function, reminiscent of AD, which was accelerated compared to littermates exposed to low-intensity sound. These changes were accompanied by distinct changes in the gut microbiome composition; *Firmicutes* was increased and *Bacteroidetes* was decreased following noise exposure [43]. Cui and colleagues then transferred the microbiome from these groups into animals that had not been exposed to noise. Mice that received FMT from the high-intensity noise group showed increased Aβ deposition in the hippocampus, indicating that the noise-induced increase in AD pathophysiology is associated with changes in the gut microbiome [43].

The inflammatory nature of the AD gut microbiome may also have deleterious effects on the entire body system and may worsen outcomes of other diseases. For instance, Soriano et al. [44] demonstrated that fecal transplants from AD mice worsen the outcome following traumatic brain injury (TBI). In this study, WT mice were subjected to controlled TBIs, followed one day later by FMT from either aged, 18- to 24-month-old 3x-Tg (AD model) mice or young WT mice. When compared to mice receiving FMT from young WT donors, recipients of FMT from aged AD donors showed increased TBI lesion size, greater impairments to motor skills, and an increase in the number of activated microglia and neuroinflammation [44]. In another demonstration of the negative effects of the AD gut microbiome, Bi et al. [45] injected mice with Lewis lung cancer cells, followed one week later by FMT from either APP/PS1 AD mice or WT littermates. The tumor sizes in recipients of FMT from AD donors were on average 40% larger compared to mice not receiving FMT. Interestingly, recipients of FMT from WT donors showed an ~46% decrease in tumor size [45]. While these investigations did not involve the study of AD symptoms themselves, they do indicate that the AD gut microbiome may worsen outcomes of other diseases.

### 2.2. Human Research

The first case study showing improvements following FMT was published in 2020 [46]; an 82-year-old male patient received a single FMT infusion from his 85-year-old wife, who served as the donor, to treat recurrent CDI, but later experienced a significant improvement in AD symptoms that continued over the following 6 months. His Mini-Mental State Examination (MMSE) score improved from a baseline of 20, indicative of mild cognitive impairment, to 29, normal cognition [46]. A second case study was published the following year. A 90-year-old female patient with AD was administered FMT from a healthy, 27-year-old female, for severe CDI [47], after which her cognitive function significantly improved across MMSE, Montreal Cognitive Assessment (MoCA) and Clinical Dementia Rating (CDR) assessment scores. Interestingly, she once again was infected by CDI, necessitating another course of FMT; her MMSE, MoCA and CDR scores improved even further following her second treatment, and she reported significantly improved mood [47].

No clinical trials have been published so far, although one was terminated early as a result of the COVID-19 pandemic [NCT03998423].

**Table 1:** AD research involving FMT.

Reference	Design	FMT	Outcomes
**Animal**			
Cui et al., 2018 [43]	**Recipients:** 3-month-old SAMP8 mice **Donors:** High-noise SAMP8 mice (HN FMT) or control SAMP8 mice (CON FMT) ***N*:** 6 per group	Delivered via oral gavage twice a week for 30 days	HN FMT recipients had increased Aβ in the hippocampus when compared to CON FMT group. Decreased expression of CLDN1 and ZO-1 in hippocampus and intestine.
Dodiya et al., 2019 [41]	**Recipients:** 7-week-old antibiotic-treated APPPS1-21 male mice **Donors:** age-matched APPPS1-21 male mice ***N*:** 9 per group	Antibiotic treatment beforehand; FMT delivered via oral gavage every day until sacrifice	Increased Aβ plaque burden and larger plaque size enlarged microglia. Higher abundance of members of *Bacteroidales* order when compared to vehicle-treated peers.
Elangovan et al., 2019 [35]	**Tg-FO:** 32-week-old 5xFAD mice, age-matched WT donors **Tg-FY:** 32-week-old 5xFAD mice, 10-week-old WT donors ***N*:** 8 per group	7 consecutive daily infusions delivered via oral gavage	**Tg-FO:** improved behavioral measures, decrease in amyloid plaque area. **Tg-FY:** significantly greater improvements than Tg-FO group along same measures.
Fujii et al., 2019 [40]	**Recipients:** 4-week-old germ-free C57BL/6N **Donors:** AD patient (AD FMT) OR healthy control (HC FMT) ***N*:** 7 per group	Single infusion	**AD FMT:** greater impairment in OLT and NORT at 55 weeks, then further deterioration at 70 weeks. **AD FMT:** reduced *Verrucomicrobia* and *Proteobacteria* compared to HC FMT group.
Sun et al., 2019 [34]	**Recipients:** APP/PS1 mice **Donors:** WT mice ***N*** = 8 per group	Antibiotics for 3 days prior; FMT delivered via oral gavage daily for 4 weeks	FMT improved behavioral outcomes, and reduced Aβ plaques and neuroinflammation. Microbiome abnormalities were corrected and butyrate levels increased.
Zhou et al., 2019 [48]	**Recipients:** male 8-week-old SD rats injected with aggregated Aβ_1-42_ **Donors:** same as donors, treated with Xanthoceraside (AXF) or sham (ASF). ***N*:** 6 per group	FMT took place 3 days after aggregated Aβ_1-42_ injection via intragastric administration once daily for 16 days	AXF group were more impaired in behavioral measures of AD than ASF group.
Kim et al., 2020 [29]	**Recipients:** ADLR^APT^ mice **Donors:** WT mice	Almost daily transfer via oral gavage for 4 months	Neurological: improvements in behavioral measures of AD, reduction in plaque area, and Aβ_40_ levels in the cortex, reduction in tau.
Shen et al., 2020 [38]	**FMT-AD:** APP/PS1 mice with AD human donor **FMT-AD-HP:** APP/PS1 mice with healthy human donor **Con-FMT-AD:** WT mice with AD human donor **N** = 5 per group	Delivered via oral gavage Incubation: 28 days (behavior tests completed weekly during incubation)	**FMT-AD:** worsened behavioral measures, increased neuroinflammation. **FMT-AD-HP:** improved behavioral measures, decreased neuroinflammation. **Con-FMT-AD:** no behavior changes but increase in neuroinflammation.
Kim et al., 2021 [39]	**Recipients:** 8-week-old C57-BL/6 **Donors:** 5xFAD mice *N*: 12	3 days on antibiotics beforehand; 5 consecutive daily infusions delivered via oral gavage	Behavioral measures were impaired, neurogenesis decreased and neuroinflammation increased. Colon inflammation increased, as did abundance of *Rikenella*. Abundance of *Barnesiella* and *Prevotalla* decreased.
Wang et al., 2021 [42]	**Recipients:** 3-month-old SPF APP/PS1 mice. **Donors:** 16-month-old APP/PS1 mice. ***N*:** 4 per group.	Antibiotic cocktail for 2 weeks prior; FMT delivered via oral gavage; 7 infusions over 7 days	Increase in Aβ deposition, inhibition of astrocyte activation. *f_Coriobacteriaceae, o_Coriobacteriales, g_Adlercreutzia, o_Erysipelotrichales*, *f_Eryslpelotrichaceae*, *f_Clostridiaceae* and *g_Clostridium* were elevated and temporally associated with Aβ plaque formation.
Bi et al., 2022 [45]	**Recipients:** female C57BL/6J mice injected with Lewis lung cancer cells **Donors:** APP/PS1 mice (AD FMT) or C57BL/6J (C57 FMT) ***N*:** 6 in each group	Intragastric administration, 3 times a week for 5 weeks 5-week incubation after FMT	AD FMT had decreased α diversity and larger tumor size. In all recipients *Prevotella*, *Mucispirillum* and *Halomonas* were elevated while *Bacteroides*, *Coprobacillus, Bifidobacterium, Faecalibacterium* and *Aggregatiacter* were decreased; changes were more severe for AD FMT than C57 FMT group.
Soriano et al., 2022 [44]	**Recipients:** 9–12-week-old WT mice with controlled cortical injury **Donors:** 9–12-week-old WT mice (FMT-young) or 18–24-month-old 3xTg-AD (FMT-AD) mice ***N*:** 5 per group	TBI one day prior to FMT, which was delivered via oral gavage for a single infusion 3 days incubation after FMT	When compared to FMT-young mice, FMT-AD mice had increased TBI lesion volume, more impaired motor function, and increased neuroinflammation. FMT-AD mice had decreased *Lachnospiraceae* and *Clostridiaceae*, increased *Eubacteriaceae*, *Oscillospiraceae* and *Muribaculaceae*.
**Human**			
Hazan, 2020 [46]	**Case study** 82-year-old male with CDI and 5-year history of AD	Single infusion	Improvements in MMSE score, memory, cognition, mood and socialization after FMT. CDI symptoms resolved. No adverse side effects.
Park et al., 2021 [47]	**Case study** 90-year-old woman with CDI, 5-year history of AD.	Two infusions three months apart, delivered via colonoscopy	Improvements in MMSE, MoCA and CDR scores. CDI resolved after both FMT treatments. Adverse side effects not reported.

Abbreviations: MMSE—Mini-Mental State Examination; MoCA—Montreal Cognitive Assessment; CDR—Clinical Dementia Rating.

## 3. Parkinson’s Disease

Parkinson’s disease (PD) is the second most common neurodegenerative disease, affecting 3–5% of the population over 65 [49]. It is characterized by gradual loss of dopaminergic cells in substantia nigra pars compacta combined with the aggregation of α-synuclein (αSyn) into Lewy bodies [49]. PD is predominantly associated with motor symptoms such as tremor or postural instability, but it also presents with several non-motor symptoms, such as sleep disturbances, psychiatric conditions and sensory symptoms [50]. PD is also associated with significant alterations in the gut microbiome [51] and gastrointestinal dysfunction [52], with constipation specifically considered one of the earliest markers of prodromal PD [53].

Many treatments exist, including exercise therapy, dopamine replacement and other pharmacologic treatment, and deep brain stimulation. However, effectiveness varies, with the most effective treatment in the early stages of PD, levodopa (L-dopa), being associated with adverse side effects such as dyskinesias and lack of impulse control [54]. Hence, there is a need for additional treatment options that allow for symptom relief without significant adverse side effects. Given the link between gut symptoms and PD, adjustment of the gut microbiome using FMT may be one such treatment. The current research is outlined below and summarized in Table 2.

### 3.1. Animal Research

One of the first studies investigating the effects of FMT in PD examined how MPTP (1-methyl-4-phenyl-1,2,3,6-tetrahydropyridine)-treated mice, a common murine model for PD, responded to FMT from healthy WT donors. Sun and colleagues reported that mice receiving FMT displayed improved motor function, increased striatal dopamine and serotonin (5-HT), and reduced dopaminergic neuron loss and neuroinflammation when compared to littermates treated with PBS [55]. Analysis of the gut microbiome showed that, at baseline, MPTP-treated mice had decreased abundance of *Firmicutes* and increased abundance of *Proteobacteria* on the phyla level when compared to WT littermates. On the order level, MPTP-treated mice had decreased prevalence of *Clostridiales* and increased abundance of *Turicibacterales* and *Enterobacteriales* when compared to WT littermates. FMT partially restored the abundance of all these groups to near WT levels in MPTP-treated recipients [55]. A second study reported that MPTP-treated mice displayed significant improvements in motor symptoms following FMT [56]. Recipient mice also showed decreased gut inflammation and neuroinflammation when compared to MPTP-treated littermates who did not receive FMT. Examination of the gut microbiome revealed increased α-diversity in MPTP mice that was corrected following FMT [56].

Further research has explored whether the gut microbiome is involved in pathogenesis, progression or severity of PD outcomes. In one study, germ-free Thy1-αSyn mice—a mouse model of PD known for accelerated α-synuclein production and decreased motor control—were administered FMT either from PD patients (PD-FMT) or healthy human (HC-FMT) volunteers [57]. Recipients of the PD microbiome displayed more severe motor deficits when compared to mice that received FMT from healthy donors [57]. Interestingly, the investigators also found that PD-FMT recipient mice had altered short-chain fatty acids (SCFAs) with increased concentrations of butyrate and propionate, but reduced acetate when compared to HC-FMT recipient mice. In fact, administration of SCFAs to the αSyn-overexpressing mice resulted in accelerated in vivo aggregation of α-synuclein and impaired motor control, implying that SCFAs are a significant element of the microbiota–gut–brain axis in PD [57].

Osteocalcin administration to 6-OHDA-induced PD mice decreases the severity of PD. When FMT was performed on WT, 6-OHDA, and osteocalcin-treated 6-OHDA mice following the depletion of gut microbiota, recipients showed varying levels of motor impairment: specifically, 6-OHDA mice showed significant motor impairments, whilst osteocalcin-treated 6-OHDA mice showed less impairment. This suggests that osteocalcin functions by modulating the gut microbiome, suggesting that targeting the microbiome with drugs may be an alternative avenue for PD treatment [58].

Overall, the animal literature to date supports FMT as a potential therapeutic avenue worthy of further exploration.

### 3.2. Human Research

The first human case study involving the use of FMT in PD was published in 2019. A 71-year-old male with a 7-year history of PD was administered FMT to treat chronic constipation, a common symptom associated with the disease. The patient experienced almost immediate relief from the symptoms of constipation, and interestingly, the tremor in his lower limbs was also significantly reduced. The resting tremor in one of his legs gradually reappeared; however, at his 3-month follow-up, the tremor was still less severe than prior to FMT [59]. More recently, Segal and colleagues reported on a series of six PD patients who were administered FMT and displayed improved motor, non-motor and constipation symptoms related to PD within 4 weeks following treatment for most patients, with minimal adverse side effects [60].

Broader clinical trials have also begun to emerge. A study of 11 PD patients reported that FMT from healthy donors, delivered via a nasoduodenal tube, was associated with noticeable improvements in PD symptomatology, both motor and non-motor [61]. A second study of 15 patients showed that FMT was associated with improved sleep and quality of life, as well as reductions in motor symptoms, anxiety and depression at a one-month follow-up [62]. A second follow-up of this same cohort at 3 months post-treatment showed enduring improvements for the 12 participants remaining in the study (three participants dropped out). Interestingly, 10 of the 15 patients in the trial received colonic FMT, whilst the remaining five were given FMT via nasoduodenal tube; the former group showed significant improvements across multiple motor and non-motor measures associated with PD compared to those receiving treatment via the colonic route, indicating that route of administration may be an important factor in FMT efficacy [62]. Adverse events reported in these studies were minimal to mild and short-lived [59,60,61,62]. Collectively, these studies demonstrate that FMT may be a viable therapy for PD patients. However, given the differences in treatment delivery and outcomes, further studies are required to elucidate the best route of delivery, general and specific outcomes, and frequency of delivery required to maintain desirable therapeutic effects.

**Table 2:** PD research involving FMT.

Reference	Design	FMT	Outcomes
**Animal**			
Sampson et al., 2016 [57]	**Recipients:** Germ-free Thy1-αSyn mice OR WT mice **Donors:** treatment-naïve PD patients (PD FMT) or healthy controls (HC FMT) ***N*:** 3–6 per group	Germ-free mice given single infusion via oral gavage.	**Neurological:** increased motor impairment when compared to HC FMT group. **GI:** less acetate, more propionate and butyrate. OTUs including *Proteus* sp., *Bilophila* sp. and *Roseburia* sp. increased; *Lachnospiraceae, Rikenellaceae* and *Enterococcus* sp. decreased.
Sun et al., 2018 [55]	**Recipients:** 8-week-old MPTP-treated C57BL/6 mice (MPTP + FMT) OR saline-treated C57BL/6 mice (NS + PD-FMT) **Donors:** C57BL/6 peers (MPTP + FMT) or MPTP-treated mice (NS + PD-FMT) ***N*:** 15 per group	Mice treated with intraperitoneal MPTP injection for 5 days. FMT delivered via oral gavage once a day for 7 days.	**MPTP + FMT:** improved motor function, increased striatal neurotransmitters, increased dopaminergic neurons, reduced glial-based neuroinflammation, and increased Firmicutes compared to controls. **NS + PD-FMT:** impaired motor function and reduced striatal neurotransmitters.
Hou et al., 2021 [58]	**Recipients:** Male C57BL/6 J mice **Donors:** 6-OHDA PD mice (6-OHDA FMT), or osteocalcin-treated 6-OHDA PD mice (OCN 6-OHDA FMT) ***N*:** 10 per group	5 weeks of antibiotics prior to 3 oral gavage FMT infusions.	**Neurological:** 6-OHDA FMT recipients had impaired motor function in rotarod and pole descent tests. OCN 6-OHDA FMT group showed relative improvements. **GI:** not reported.
Zhang et al., 2022 [56]	**Recipients:** 11–13-week-old MPTP-treated C57BL/6 mice **Donors:** 6–8-week-old C57BL/6 mice	5 weeks of MPTP injection followed by 14 FMT infusions.	**Neurological:** improvements in behavioral tests; protection against SN neuronal damage, reduced neuroinflammation. **GI:** decreased inflammation in the intestine; *Anaerostipes*, *Bifidobacterium*, *Turicibacter*, *ASF356* and *Ruminococcus* decreased; *Blautia* increased.
**Human**			
Huang et al., 2019 [59]	Case study **Recipient:** 71-year-old male PD patient **Donor:** 26-year-old healthy male	FMT delivered as 200 mL of solution once per day for 3 days via transendoscopic enteral tube. Follow-up appointments were at 1 week, 2 months, 3 months	UPDRS III scores improved from 46 to 32, then slowly rose to 44 over 3 months. Resting tremor in legs almost disappeared at 1 month, but slowly reappeared. Phyla *Firmicutes* increased, *Proteobacteria* and *Bacteroidetes* decreased. No adverse side effects.
Xue et al., 2020 [62]	Preliminary clinical trial **Recipients:** **Donors:** healthy human donors (18 to 24 years old) ***N***: 15	Single infusion delivered via nasoduodenal tube (n = 5) or colonic delivery (n = 10). Follow-up between 1–12 months	Mean UPDRS III scores decreased from 41.75 to 24 by month 3. PSQI, HAM-D, HAM-A, PDQ-39, and NMSQ also improved. **Adverse side effects:** 5 patients had mild AEs like diarrhea, abdominal pain or flatulence.
Kuai et al., 2021 [61]	Prospective clinical study **Recipients:** PD patients **Donors:** donor bank ***N*:** 11	Low-carb diet and fasting required before single infusion, delivered via nasoduodenal tube. Follow-up at 12 weeks.	UPDRS, NMSS, PAC-QOL, and Wexner constipation scores improved. Specific microbiota abundances fluctuated over time after FMT. **Adverse side effects:** mild diarrhea, abdominal pain, venting, flatulence, nausea and throat irritation occurred; however, all were mild and did not interrupt treatment.
Segal et al., 2021 [60]	Case series of 6 PD patients. **Donors:** 38-year-old male and 50-year-old male, both healthy	Macrogel bowel prep used prior to single FMT, delivered via colonoscopy. Follow-up at 2, 4, 8, 12, 16, 20 and 24 weeks	Significant improvements in UPDRS III, NMSS and Wexner constipation scores for at least 5/6 patients. **Adverse side effects:** one patient had vasovagal pre-syncope requiring observation.

Abbreviations: GI—gastrointestinal; UPDRS—Unified Parkinson’s Disease Rating Scale; PSQI—Pittsburgh Sleep Quality Index; HAM-D—Hamilton Depression Rating Scale; HAM-A—Hamilton Anxiety Rating Scale; PDQ-39—39-item Parkinson’s Disease Questionnaire; NMSQ—Non-Motor Symptoms Questionnaire; NMSS—Non-Motor Symptoms Scale; PAC-QOL—Patient Assessment of Constipation-Quality of Life.

## 4. Multiple Sclerosis

Multiple Sclerosis (MS) is the single most common demyelinating disease, characterized by gradual loss of myelination throughout the nervous system [63]. Primary symptoms directly related to demyelination include weakness, sensory loss and impaired balance, whilst secondary and tertiary symptoms, such as urinary tract infections and social isolation, develop as a consequence [64]. MS is often considered to progress through multiple stages, with gradual worsening of symptoms over time. In most cases, MS first occurs as a clinically isolated syndrome (CIS) consisting of various neurological symptoms that present for a few days, usually followed by subsequent periods of relapse and remission characteristic of relapsing-remitting MS (RRMS). Within 10–15 years, RRMS typically progresses to secondary-progressive MS (SPMS), as symptoms shift from a pattern of relapses to gradual progression without remission [65]. A small proportion of patients develop primarily progressive MS (PPMS) from onset without relapses early in the disease [65]. It is generally accepted that MS is an autoimmune disease, given that localized invasion of immune cells and cytokines in the CNS is considered a primary cause of progressive damage [66].

While there is a genetic component to the disease, environmental factors such as smoking, geographical location, vitamin D intake, and infection with Epstein–Barr virus, suggest that other factors play a role in its pathogenesis [67]. MS patients often present with specific differences in gut microbiome composition, including a decrease in the genus *Prevotella* or increase in *Akkermansia* [68,69], suggesting an interaction between the gut and brain in MS. 

Currently, there is no cure for MS and most therapeutics are aimed at providing symptomatic relief [64], with particular emphasis on treating primary symptoms as they appear to prevent the subsequent development of secondary or tertiary symptoms [70]. Therefore, potential new disease-modifying treatments are needed. Current research into FMT for MS is outlined below and summarized in Table 3.

### 4.1. Animal Research

Multiple animal studies have emerged involving FMT and MS, predominantly using the experimental autoimmune encephalomyelitis (EAE) model commonly used to replicate MS. In one, EAE mice were administered FMT from WT donors. Recipients showed significant improvements in EAE-related clinical scores and had fewer infiltrating cells in their spinal cords when examined with immunofluorescent imaging. Additionally, FMT-treated mice had microbiomes similar to WT donors but distinct from EAE controls [71]. Intriguingly, mice received five FMT treatments over a period of 9 days and were sacrificed two days following the final treatment. These results demonstrate the acute nature of FMT and the fact that a short course of treatment was capable of repopulating the gut microbiome with beneficial bacteria. Li and colleagues reported that EAE mice treated with FMT from WT littermates showed significantly delayed onset and reduced clinical symptoms, as well as a slowed progression of EAE. These changes were associated with reductions in demyelination as well as astrocytic and microglial activation [72].

Liu and colleagues made a remarkable discovery when they used FMT from EAE mice that were at the peak of EAE disease. Transplanting this fecal matter into naïve animals who were then treated to induce EAE resulted in the amelioration of disease in recipient mice as compared to FMT from healthy naïve mice [73]. Further investigation revealed that the changes were driven by micro-RNA-30d (miR-30d) present in the gut microbiota [73]. The authors report that the increase in miR-30D resulted in increased abundance of *Akkermansia muciniphila*, an anti-inflammatory bacterium that increases regulatory T-cells (Tregs), which in turn ameliorated the symptoms of MS before it could progress [73]. This result is interesting, as it is counterintuitive and contradicts a separate study that used a mouse model of RRMS known to develop spontaneous EAE in ~80% of animals. Berer and colleagues used fecal matter from a human MS-discordant monozygotic twin pair as donor material, and showed that mice that received FMT from the MS-affected twin were more likely to develop EAE than littermates that were administered FMT from the unaffected twin [74]. The interaction between the gut microbiome, miR-30D, and MS symptoms is therefore a potential area of interest and must be further investigated, as it may hold a key to therapeutic intervention. 

One final study involved transferring FMT from either MS patients or healthy household members into germ-free mice [75]. Six weeks following transplantation, mice were immunized to induce EAE and results showed that disease in recipients of FMT from MS patients progressed faster compared to littermates who were administered FMT from healthy controls, accompanied by an upregulation of genes related to immune response. This study suggests that the gut microbiome of MS patients contributes to a pro-inflammatory state that further exacerbates the disease [75].

### 4.2. Human Research

In 2011, a case series was published by Borody and colleagues, detailing improvements seen in three MS patients following the administration of FMT to treat constipation [76]. One patient was a 30-year-old male with comorbid MS and trigeminal neuralgia who had severe leg weakness necessitating wheelchair use. Following five FMT infusions, the patient regained the ability to walk and experienced remission lasting at least 15 years [76]. The second, a 29-year-old male with ‘atypical’ MS, presented with paresthesia and leg muscle weakness. Following 10 FMT infusions, the patient experienced gradual resolution of motor, urinary and gastrointestinal function that lasted at least 3 years [76]. Finally, an 80-year-old female with ‘atypical’ MS who presented with difficulty walking, gradually became asymptomatic over two years after receiving five FMT infusions [76].

Similar case studies have since emerged. In 2018, a 61-year-old female with SPMS was administered a single infusion of FMT to treat recurrent episodes of CDI. She showed immediate stabilization of her Expanded Disability Status Scale (EDSS) score (a method of quantifying disability and monitoring changes in the level of disability in MS), which had previously been progressively increasing [77]. The patient experienced a subsequent 10 years of stability, within which her functionality somewhat improved [77]. Similarly, a 52-year-old female with a 20-year history of RRMS was also administered FMT to treat recurrent CDI [78]. She showed improvements in GI symptoms within a week, but after a year had also shown improvements in muscle strength and a marginal improvement in EDSS scores [78]. Engen and colleagues describe the case of a 48-year-old male with a two-year history of RRMS and difficulty walking. Following 10 FMT infusions, his walking significantly improved across a range of metrics, including gait, cadence and walking speed [79]. These improvements were still present at the 12-month follow up with no relapses during that period. Notably, the authors discovered that the abundance of a butyrate-producing bacteria, *Faecalibacterium prausnitzii*, SCFA abundance, and α-diversity in the microbiome of this patient were increased following FMT [79].

One clinical trial was terminated during the recruitment phase due to insufficient enrolments. The authors reported no significant improvements in MRI, EDSS or plasma cytokine levels, although the lack of statistical power resulting from a small sample size may have obscured the outcomes of the study. Despite this, the authors were able to confirm that FMT was safe for their participants, and that the transplant resulted in the correction of the abnormally low α-diversity observed in the patients [80].

**Table 3:** MS research involving FMT.

Reference	Design	FMT	Outcomes
**Animal**			
Berer et al., 2017 [74]	**Recipients:** GF spontaneous RRMS mice **Donors:** MS patient (MS FMT, n = 24) or their healthy twin (HC FMT, n = 23)	One infusion delivered via oral gavage	**Neurological:** recipients of MS FMT had increased incidence of spontaneous MS, reduced IL-10 production when compared to HC FMT mice. **GI:** reduced abundance of *Sutterella* in MS FMT recipients.
Cekanaviciute et al., 2017 [75]	**Recipients:** GF C57BL/6 mice **Donors:** MS patients (MS FMT, n = 11) or healthy household members (HC FMT, n = 9)	2 weeks of antibiotic cocktail beforehand. FMT delivered via oral gavage every 2–3 days for 2 weeks. EAE immunization 6 weeks later	**Neurological:** MS FMT mice showed significantly increased EAE disease scores, increase in genes expressed by microglia. **GI:** decrease in *Sutterella*, increase in *Ruminococcus*. **Other:** decreased IL-10+ Treg induction
Liu et al., 2019 [73]	**Recipients:** C57BL/6J mice **Donors:** EAE mice ***N*:** 7–10 per group	FMT delivered via oral gavage 6, 4, and 2 days before EAE immunization. Heat-killed FMT was also used to investigate role of micro-RNA	**Neurological:** decreased EAE score, reduced demyelination, less axonal loss. Protective mechanism was tied to miR-30D after heat-treated FMT produced similar ameliorating effect in subsequent experiments.
Wang et al., 2021 [71]	**Recipients:** 6–8-week-old female C57BL/6 EAE mice, n = 8 **Donors:** C57BL/6	FMT delivered via oral gavage every 2nd day for 9 days	**Neurological:** improved EAE score, less spinal inflammation, and fewer infiltrating cells in their spines. **GI:** FMT restored proportion of *Firmicutes*, *Proteobacteria*, *Bacteroidetes*, and *Actinobacteria*.
**Human**			
Borody et al., 2011 [76]	Case series **(1)** 31-year-old man with MS; **(2)** 29-year-old man with atypical MS; **(3)** 80-year-old woman with atypical MS	**(1)** 5 infusions, 15-year follow-up **(2)** 10 infusions, 3-year follow-up **(3)** 5 infusions, 2-year follow-up	**Neurological:** progressive improvement in walking ability and urinary function in all patients; patient 3 appeared asymptomatic. **GI:** all 3 patients showed improvements in constipation.
Makkawi et al., 2018 [77]	Case study **Recipient:** 61-year-old woman with MS **Donor:** her partner	Single infusion via rectal enema. 10 years follow-up.	**Neurological:** EDSS score stabilized; improvements in Functional System scores and Modified Multiple Sclerosis Functional Composite scores over 10 years. **GI:** resolution of CDI.
Engen et al., 2020 [79]	Case study **Recipient**: 48-year-old male with RRMS **Donors:** multiple donors	Standard bowel prep prior to FMT delivered via rectal enema. 10 infusions in weeks 1–3; 5 during weeks. Follow-up 3, 13, 26, 39 and 52 weeks.	**Neurological:** BDNF increased from below normal range at baseline. Gait metrics improved after both FMT treatment rounds. **GI:** *Faecalibacterium prausnitzii* increased over the course of the study. Propionate, butyrate, total SCFA were increased at 13 and 39 weeks.
Garcia-Rodriquez et al., 2020 [78]	Case study **Recipients:** 52-year-old woman with RRMS patient	Oral lyophilized FMT. Follow-up at 1 year.	**Neurological:** horizontal nystagmus, 2/5 muscle strength in right limbs, EDSS score of 8.5 at baseline. FMT associated with increased strength and EDSS score of 8 after 1 year. **GI:** resolution of CDI after 1 week.
Al et al., 2022 [80]	Clinical trial **Recipients:** RRMS patients **Donors:** 2 donors ***N*:** 9	FMT delivered via rectal enema. 6 patients had 8 monthly infusions; 1 patient had 5; 2 patients had 2. Follow-up varied.	**GI:** Donor 1 recipients had enriched *Hungatella hathewayi* and decreased *Faecalibacterium, Subdoligranulum* and *Blautia* after FMT; donor 2 recipients had increases in *Phascolarctobacterium succinatutens*. **Adverse side effects:** 1 patient developed hives that resolved without treatment.

Abbreviations: GI—gastrointestinal; EDSS—Expanded Disability Status Scale; SCFA—short-chain fatty acids.

## 5. Amyotrophic Lateral Sclerosis

Amyotrophic lateral sclerosis (ALS) is a neurodegenerative disease associated with progressive loss of upper and lower motor neurons, leading to gradual loss of motor function accompanied by cognitive and behavioral changes that are within the spectrum of frontotemporal dementia [81]. The exact mechanism underlying the disease is not well known, but toxic protein aggregation, mitochondrial dysfunction, excitotoxicity and other cellular and molecular processes have been implicated with its development [82]. There is currently no cure, and the disease is typically fatal within 2–4 years of diagnosis. Three drugs are currently approved for the treatment of ALS but are only able to extend life expectancy by 3–6 months or slow the rate of decline in patients. Other treatments are aimed at relieving individual symptoms of the disease [83].

As with other neurodegenerative diseases described, ALS is associated with altered gut microbiome composition in humans and animal models alike [5,84]. There is hope that the gut microbiome may therefore be an additional target in the search for disease-altering treatments. The current human research is outlined below and summarized in Table 4. No studies investigating the effect of FMT in animals have, to our knowledge, been reported.

### Human Research

We have only been able to identify a single report of a 48-year-old female with ALS who experienced constipation, gradual muscle fasciculation and amyotrophy in her left leg, later spreading to her right leg and arms [85]. She was placed on a long-term treatment plan of washed microbiota transplantation (WMT)—a modified form of FMT—and reported improved bowel function and ALS symptoms. Several months later, she was prescribed antibiotics due to a scalp injury and her condition rapidly deteriorated. However, following a re-introduction of the WMT routine, her ALS symptoms once again stabilized [85].

No other research involving FMT in ALS has yet been published, but a clinical trial is underway [86].

**Table 4:** ALS research involving FMT.

Reference	Design	FMT	Outcomes
**Human**			
Mandrioli et al., 2019 [86]	Randomized clinical trial (ongoing) **Recipients:** early-stage ALS patients **Donors:** *N*: 42	Pre-treatment: not specified Administration route: nasojejunal tube Repetition: repeated at 6 months	N/A
Lu et al., 2022 [85]	Case study **Recipients:** 48-year-old woman with ALS **Donors:** not specified *N*: 1	Pre-treatment: washed microbiota transplant (altered FMT procedure) Administration route: mid-gut or colonic transendoscopic tube Repetition: Repeated infusions every 2 months. Brief period of antibiotic treatment at midpoint.	**Neurological:** from the first treatment, improvements were seen in ALS Functional Rating Scale-Revised, 40-item ALS assessment questionnaire, muscle tone and modified Ashworth spasticity scale. Repeated treatments resulted in plateau of symptoms. **GI:** constipation relieved; reconstruction of gut microbiome after antibiotic challenge. Adverse side effects: none.

## 6. Conclusions

Despite extensive research over a number of decades, disease-altering treatments for neurodegenerative diseases are not yet available. When they do exist, treatments are generally aimed at alleviating symptoms, and may come with significant adverse side effects, limiting their use. There is therefore a desperate need for new treatment options that improve quality of life or modify the disease itself in a meaningful way. The evidence summarized in this review suggests that FMT is a potential treatment for these diseases that has, to date, not been explored.

It is important to note that this specific area of research is still in its infancy and there are still several limitations regarding the current pool of research. For instance, while case studies are valuable for providing inspiration for future studies, and animal studies can provide further insight, clinical trials are still either very limited or lacking entirely. Additionally, FMT protocols are rarely standardized across multiple studies, and it is therefore difficult to make meaningful inferences on a meta scale. As noted previously in the section on PD, different delivery routes (e.g., nasoduodenal tube or colonic delivery) can produce significantly different outcomes [62]. Some studies may administer antibiotics beforehand, others might not; some studies may use a single FMT infusion, others may use multiple. Guidelines do exist to aid researchers in selecting donors; however, standardized protocols are still not available [87]. Given this absence, it would be beneficial for future research to either establish standard procedures or examine the impact of factors such as those mentioned, so that comparison between studies may become possible.

Despite these limitations, the evidence discussed in this review does suggest that gut microbiome modification through FMT may be a novel treatment for AD, PD, MS and ALS that should be investigated in more depth. At the very least, it appears to provide some relief from symptoms with minimal (if any) adverse side effects; this is incredibly valuable in an area where treatments are either missing or limited in their long-term efficacy. Therefore, we eagerly await the emergence of further research, particularly, clinical trials.

**Note:** Whilst this manuscript was under review, Elangovan, Borody and Holsinger published their manuscript [88] that was referred to as a preprint [35] in Section 2.1 above.

## Data Availability

Not applicable.
